# Comparative Efficacy of Spinal Cord Stimulation in the Management of Acute Pain and Chronic Pain Related to Failed Back Surgery Syndrome: A Systematic Review and Meta-Analysis of Randomized Controlled Trials

**DOI:** 10.7759/cureus.71132

**Published:** 2024-10-09

**Authors:** Jaden Y Fang, Hideaki Yamamoto, Adam N Romman, Aristides Koutrouvelis, Satoshi Yamamoto

**Affiliations:** 1 Anesthesiology, University of Texas Medical Branch (UTMB), Galveston, USA; 2 Biological Sciences, University of California San Diego, San Diego, USA

**Keywords:** acute pain management, chronic pain management, failed back surgery syndrome (fbss), postoperative pain relief, spinal cord stimulation (scs)

## Abstract

Spinal cord stimulation (SCS) is a well-established treatment for chronic pain. However, its potential in acute pain management requires further investigation. The goal of this review is to assess and compare the effectiveness of SCS for managing acute postoperative pain against chronic pain associated with failed back surgery syndrome (FBSS). A comprehensive search of databases identified randomized controlled trials (RCTs) that examined SCS for both acute and chronic pain associated with FBSS. Pain relief was measured using the Visual Analog Scale (VAS) and Numeric Rating Scale (NRS). Study quality was evaluated using the Jadad score and Cochrane risk of bias tool. Evidence suggests that SCS significantly reduces acute pain, achieving over a 50% reduction in VAS scores. For chronic pain associated with FBSS, SCS demonstrated substantial efficacy, with a mean reduction of -2.45 on pain scales compared to baseline. When compared to optimal medical management (OMM), SCS was more effective, showing a mean reduction of -1.17 in pain scores for FBSS. Overall, SCS offers significant benefits in managing chronic pain, particularly in FBSS, by reducing pain intensity and opioid use. While the initial findings for acute pain relief are promising, further high-quality RCTs are needed to better understand SCS's role in preventing the transition from acute to chronic pain. Continued research into optimizing patient selection and stimulation parameters will be essential to improve therapeutic outcomes in both acute and chronic pain management.

## Introduction and background

Spinal cord stimulation (SCS) has emerged as a revolutionary therapy in the management of intractable pain, offering hope to patients with chronic conditions such as failed back surgery syndrome (FBSS). SCS is a therapeutic intervention used to manage chronic pain, particularly in cases where other treatments have failed. This technique involves the implantation of electrodes that deliver electrical signals to the spinal cord, aiming to modulate pain perception. Having conceptualized the gate control theory [1] of pain, SCS has evolved significantly, with clinical reports highlighting its efficacy mainly in chronic pain scenarios [2]. The advent of SCS marked a paradigm shift in pain management. Its application in chronic pain, especially FBSS, has been well-documented, with numerous studies demonstrating substantial pain relief and improved quality of life [3]. Characterized by persistent or recurring low back and/or leg pain following one or more spine surgeries, FBSS often leaves patients with limited treatment options. However, SCS offers a modality that can modulate pain signals within its mechanism, providing a respite from the relentless pain or discomfort associated with this condition. In contrast, the investigation into SCS in the context of postoperative pain following spinal surgery remains underdeveloped. Notwithstanding the recent empirical evidence concerning the efficacy of SCS in mitigating postoperative pain during spinal surgery in a rodent model, as well as the fact that inadequately addressed postoperative pain can precipitate chronic pain conditions such as FBSS, extended hospital admissions, and a series of detrimental repercussions, the paucity of clinical studies indicates a potentially beneficial application of SCS in this area [4-6]. More specifically, postoperative pain after spinal surgery is widely recognized as a formidable challenge [7]. Thus, early intervention with SCS could potentially alter the trajectory of acute pain, preventing its transition to a chronic state.

The initially proposed mechanism by which SCS exerts its effects is hypothesized to involve the modulation of nociceptive pathways across various tiers of the nervous system [8-10]. By delivering electrical impulses to the spinal cord, SCS can inhibit pain transmission and induce analgesia. This neuromodulation technique has been refined over the years, with advancements in electrode design and stimulation parameters enhancing its therapeutic efficacy [11]. Through the administration of electrical impulses to the spinal cord, SCS possesses the capacity to inhibit the transmission of pain and facilitate analgesia. Advances in SCS waveforms and programming have increased the efficacy for chronic pain control with novel mechanisms of action postulated including modulation of neurotransmitters, wide-dynamic-range neurons, glial cells, and other neural targets within the brain [12]. These hypotheses are mainly tied to the administration of chronic pain [13]. In comparative terms, the utilization of SCS in the management of acute pain emerges as a domain that warrants extensive research. The potential for SCS to mitigate acute postoperative pain and reduce reliance on opioid analgesics provides a compelling rationale for its incorporation into perioperative care paradigms; however, the foundational evidence supporting this application remains in its infancy [14]. As a result, while SCS has established itself as a core methodology in the management of chronic pain conditions such as FBSS, its application within the domain of acute pain management, especially regarding postoperative intense pain, signifies a promising avenue for scholarly inquiry. Moreover, despite the existence of systematic reviews that have evaluated the efficacy of SCS in the context of FBSS through case series and observational studies, there exists a notable absence of systematic reviews within the current literature concerning a comprehensive meta-analysis that specifically addresses randomized controlled trials (RCTs) related to SCS in the framework of FBSS [15,16]. It is noteworthy that meta-analyses have been performed concerning SCS in the broader context of chronic pain [17]. Therefore, it is essential to systematically scrutinize the extant evidence to determine the clinical efficacy of SCS associated with FBSS and its pertinence to acute pain contexts.

The objectives of this investigation are to consolidate contemporary evidence and elucidate the comparative effectiveness of SCS within these two domains while also seeking to explore the prospective applications of SCS in the management of postoperative pain following spinal surgery in future clinical studies. Concurrently, this study is undertaken with a pronounced emphasis on contemporary discoveries related to acute pain, as opposed to the traditional employment of SCS within the framework of FBSS, encompassing an updated meta-analysis of the relevant RCTs.

## Review

Methods

Search Strategy

We conducted a comprehensive search of prominent electronic medical information repositories, encompassing PubMed, MEDLINE, Google Scholar, ScienceDirect, BMJ databases, and Cochrane databases, to identify cohort studies from inception to July 2024 pertaining to acute pain and SCS, as well as chronic pain resulting from FBSS and SCS, in collaboration with a professional librarian. To guarantee the thoroughness of our search, the reference lists of the sourced articles were meticulously scrutinized. Two authors (JF and HY) independently undertook the data extraction process. Any discrepancies that arose were resolved through a consensus mechanism among the authors. Following the search, two independent reviewers evaluated the articles to ascertain their adherence to the inclusion criteria, the verification of human subjects, and the requirement of being published in the English language. This systematic review has been duly registered with the International Platform of Registered Systematic Review and Meta-analysis Protocols (INPLASY) (registration number: INPLASY202470082). The search flow diagram strictly follows the Preferred Reporting Items for Systematic Reviews and Meta-Analyses (PRISMA) guidelines.

Study Selection

Acute pain is characterized by the International Association for the Study of Pain (IASP) as a normal, predicted physiological response to adverse stimuli, which may include chemical, thermal, or mechanical influences, and is generally elicited by a distinct inciting event such as traumatic injury, surgical procedures, or acute medical conditions, typically subsiding within a brief temporal frame, whereas chronic pain is delineated as an "unpleasant sensory and emotional experience associated with, or resembling that associated with, actual or potential tissue damage" typically lasting longer than three months [18,19]. Two separate searches were conducted. The first search included studies that described patients with acute pain within the context of SCS application with search terms "acute pain" and "SCS." The second search included studies with "chronic back and leg or lower limb pain" specifically secondary to FBSS, within the context of SCS application with search words "chronic pain," "Failed Back Surgery Syndrome," and "SCS." Within the scope of chronic pain, records that measured the pain scale during a period of three to six months after the application of SCS were integrated. The studies selected for inclusion were required to explicitly articulate either the duration or the onset of pain as a characteristic of the targeted patient demographic within RCTs. In the event that the research investigated various stimulation frequencies, the minimum frequency was included in the analysis. Case reports, case series, studies with peripheral nerve stimulation and non-randomized cohort studies, and clinical trials that involved participants who had previously undergone SCS prior to the initiation of clinical trials were systematically excluded from consideration. The research merely comparing the stimulation frequency of SCS in its efficacy was likewise excluded.

Risk of Bias and Methodological Quality Assessment

To evaluate the methodological rigor of studies incorporated in this analysis, the "risk of bias" instrument formulated by the Cochrane Collaboration was employed. This tool assesses studies according to criteria that encompass the utilization of single- or double-blind designs, randomization processes, potential selection bias concerning outcomes, data completeness, and outcome evaluations. The risk of bias for each study was categorized as low, high, or unclear in accordance with established criteria. Furthermore, for randomized trials, the Jadad score was applied to appraise the methodological quality of RCTs. This tool assesses studies based on three fundamental criteria: randomization procedures, blinding methodologies, and documentation of withdrawals and dropouts. The scoring system ranges from 0 to 5, wherein elevated scores signify superior quality.

Patient Population, Data Collection, and Statistical Analysis

Demographic and clinical characteristics of patients from published series and patients with pain were compared by summary. Our principal efficacy outcome encompassed pain alleviation. To evaluate pain, we employed the Visual Analog Scale (VAS; 0-10 cm) as well as the Numeric Rating Scale (NRS; 0-10). We standardized both the VAS and the NRS, subsequently converting the VAS by normalizing pain scores through division by 10. In instances where there exist several temporal assessments of outcomes within the timeframe of three to six months, the initial follow-up outcomes were incorporated into the analytical framework. For continuous outcomes in the meta-analysis, the effect was computed as the mean difference (MD), accompanied by a confidence interval (CI) and a corresponding p-value. An MD value of 0 was indicative of a neutral effect, while a 95% CI that intersected 0 was deemed non-significant, with p-values considered significant if they were less than 0.05. The magnitude of the effect for quantitative outcomes was derived from the mean, standard deviation, and population size associated with each individual study. The selection between common and random effects analysis was made in favor of the latter due to the observed heterogeneity of the interventions. Heterogeneity is influenced by clinical, methodological, and statistical factors such as patient population. When the quantity of studies providing usable data for a specific outcome reached three, a common effects analysis was conducted, owing to the ambiguity surrounding the variance between studies. For outcomes represented solely by one or two studies, the data were conveyed through a narrative description. A heterogeneity assessment was conducted, encompassing the comparison of Cochrane's Q against the degrees of freedom (df), the variance of true effects (T²), and the actual difference in effect size (I²), although this information was not utilized to determine the analytical approach (fixed versus random) to be employed. The software employed for this analysis was the R statistical package (R Foundation for Statistical Computing, Vienna, Austria, https://www.R-project.org/). This report was composed in accordance with the PRISMA checklist.

Results

SCS on Acute Pain

**Figure 1:**
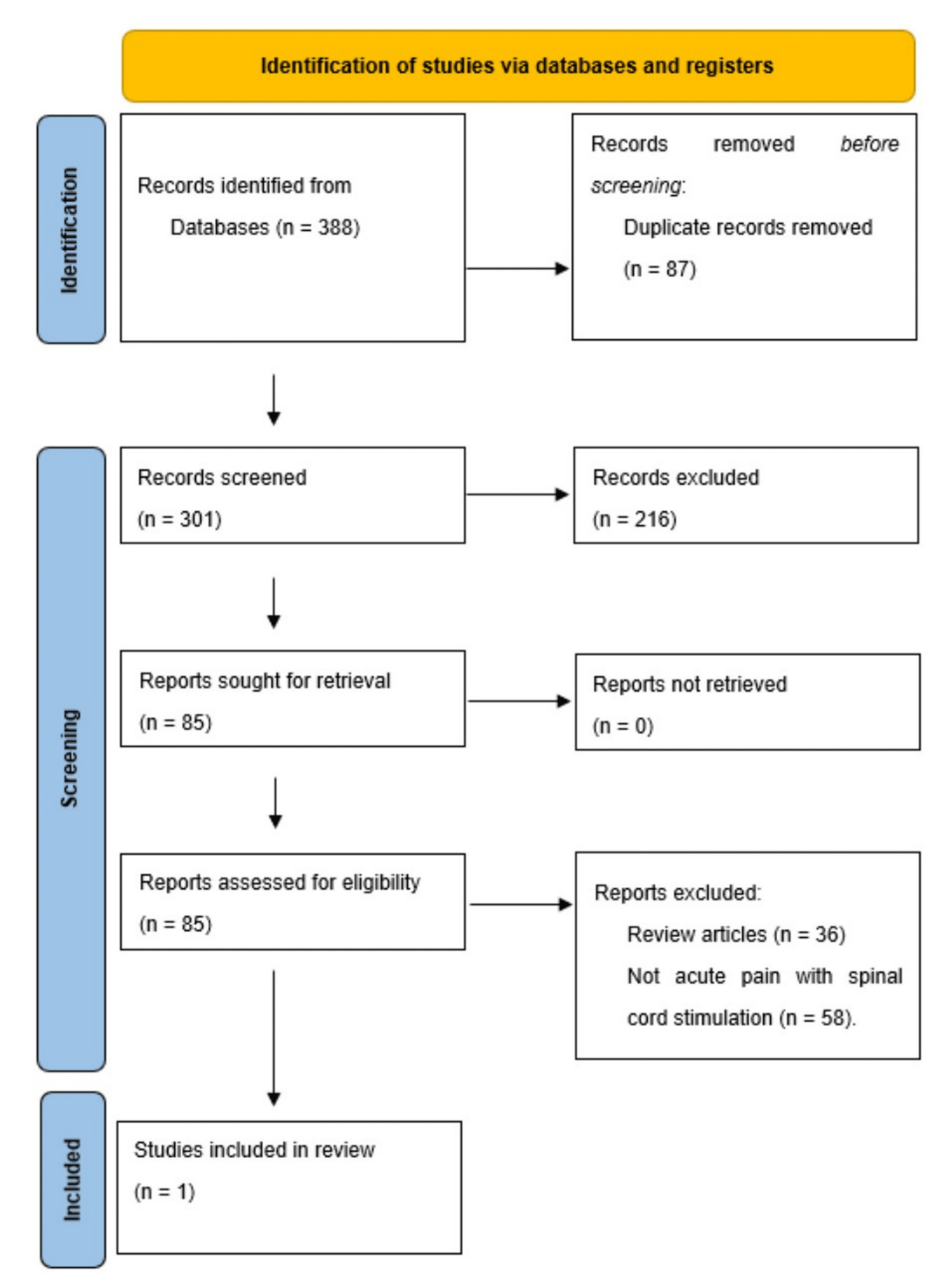
PRISMA flow diagram of the study selection process for acute pain and SCS PRISMA: Preferred Reporting Items for Systematic Reviews and Meta-Analyses; SCS: spinal cord stimulation

Study identification and inclusion: In the examination of SCS pertaining to acute pain, an initial identification of 388 records was made, of which 303 were subsequently excluded based on pertinent exclusion criteria derived from the titles and abstracts. The residual 85 records underwent a meticulous screening process and were rigorously evaluated against the relevant exclusion criteria concerning systematic reviews, chronic pain, cranial stimulation, and animal models. A singular full-text article was subjected to an assessment of eligibility (Figure 1) [20]. 

Efficacy of SCS on Acute Pain

The objective of the study was to evaluate and compare the safety and efficacy of temporary SCS against pulsed radiofrequency (PRF) in the management of postherpetic neuralgia (PHN). The investigation encompassed a sample of 44 participants who were randomly allocated to either the SCS or PRF intervention group for a treatment period lasting one day. Evaluations were carried out at multiple time intervals: prior to surgery, followed by assessments at one week, one month, three months, and six months postoperatively. The findings of the study revealed a significant reduction in VAS scores for both the SCS and PRF groups as early as one week following the intervention. More specifically, the average postoperative VAS score was recorded as 2.0 in the SCS group indicating that SCS facilitated a more substantial alleviation of pain intensity relative to PRF. The enhancements in pain reductions associated with SCS were not only statistically significant immediately following treatment but were also sustained throughout the six-month follow-up period. This observation implies that SCS may confer an enduring analgesic effect.

SCS on Chronic Pain Secondary to Failed Back Surgery

**Figure 2:**
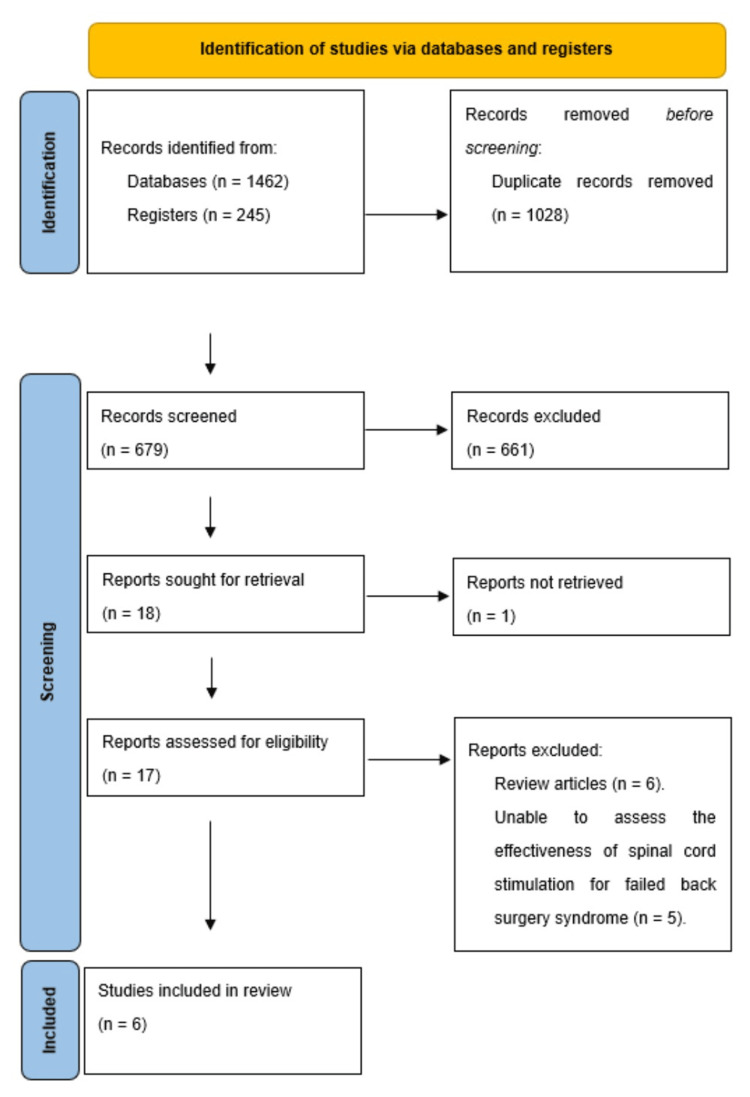
PRISMA flow diagram of the study selection process for chronic pain secondary to FBSS and SCS PRISMA: Preferred Reporting Items for Systematic Reviews and Meta-Analyses; FBSS: failed back surgery syndrome; SCS: spinal cord stimulation

Study identification and inclusion: In the comprehensive systematic review addressing SCS for the management of chronic back and lower limb pain related to FBSS, a total of 1707 records were originally identified, of which 1028 were later excluded owing to duplication. A total of 679 articles have undergone scrutiny through the examination of their titles and abstracts, leading to the exclusion of 661 articles; of the remaining 18 articles, one record was excluded as irretrievable, and 17 constitute the definitive corpus that is qualified for comprehensive evaluation of the complete manuscripts. Due to a lack of pertinent outcome measures or baseline assessments, 11 trials were excluded. Thus, six full-text articles were evaluated for eligibility (Figure 2) [21-26]. 

The attributes of the studies incorporated in this review are presented in Table 1. To appraise the methodological quality and assess the risk of bias of the included studies, both the Cochrane risk of bias assessment and the Jadad score were employed. Among the six studies selected, four were classified as high quality (score ≥3) while two were designated as low quality (score <3) according to the Jadad scale (Table 2). In the context of the Cochrane risk of bias assessment, three studies were categorized as presenting a low risk of bias, whereas three were classified as exhibiting a moderate risk of bias (Table 3).

**Table 1:** Characteristics of the included studies The review encompasses six randomized controlled trials conducted in Norway, Spain, the USA, and multiple multinational settings. These studies involved a total of 342 patients suffering from various forms of chronic pain, including FBSS, chronic radicular pain, and neuropathic pain. Two studies focused on patients with FBSS, while one trial restricted its participants to those with chronic radicular pain following lumbar spine surgery. High-frequency SCS was investigated in three studies. Two studies compared conventional low-frequency SCS to high-frequency options. One study used a burst stimulation technique. The studies employed varied follow-up times, ranging from one to 24 months. SCS: spinal cord stimulation; FBSS: failed back surgery syndrome; VAS: Visual Analog Scale; NRS: Numeric Rating Scale; PD-Q: PainDETECT Questionnaire; ODI: Oswestry Disability Index; NPRS: Numeric Pain Rating Scale; SF-36: Short Form-36; PCS: Physical Component Score

Study name	Country	Type of study	Subjects	Treatment level	Frequency	SCS device parameters	Setting	Crossover study	Funding source	Adverse effects	Follow-up timepoints	Outcomes
Al-Kaisy et al., 2018 [21]	Multinational	Prospective, randomized, sham-controlled, double-blind, crossover	24 patients with FBSS	T7-T10	High frequency	1200 Hz, 3030 Hz, 5882 Hz	Multiple centers	Yes	Not specified	Not specified	3 months	VAS, back pain score
Bolash et al., 2019 [22]	USA	Prospective randomized controlled	38 patients with chronic pain in FBSS	T8-T11	High frequency	High-frequency (10 kHz) SCS	Multiple centers	No	Stimwave Technologies Incorporated	Lead migration	1, 3, 6 months	Back pain, leg pain VAS
De Andres et al., 2017 [23]	Spain	Prospective, randomized, blind	29 patients with pain and disability due to FBSS	T8-T9	Conventional vs high frequency	40 Hz and 10000 Hz	Hospital	No	None	Not specified	3, 6, 12 months	NRS (pain), PD-Q, ODI
Hara et al., 2022 [24]	Norway	Randomized clinical trial	84 patients with chronic radicular pain after lumbar spine surgery	Epidural space at the T9-T10 level	Conventional	40 Hz burst SCS	Hospital	Yes	Liaison Committee for Education, Research, and Innovation (Central Norway)	Surgical revision of device	3 months	ODI, NRS, EuroQol 3L index score, steps per day
Kumar et al., 2007 [25]	Multinational	Prospective randomized controlled	88 patients with neuropathic pain	Not specified	Conventional	Neurostimulation system with mean 49 Hz	Multiple centers	Yes	Medtronic	Worsening of preexisting condition	1, 3, 6 months	Leg pain, back pain (VAS), quality of Life, ODI
Rigoard et al., 2019 [26]	Multinational	Multicenter randomized controlled	79 patients with predominant back pain in FBSS	Not specified	Conventional	Not specified	Multiple centers	Yes	Medtronic	Implant site infection, surgical revision of device	6, 12, 24 months	Reduction in low back pain, back/leg pain intensity (NPRS), ODI, SF-36, PCS

**Table 2:** The Jadad quality assessment for the included studies All studies were assessed for randomization, blinding, and participant withdrawals. Four studies were evaluated as "high quality." These studies demonstrated robust methodologies with well-implemented randomization, appropriate blinding, and clear reporting of withdrawals. Two studies were rated as "low quality" due to limitations in blinding procedures, which could introduce bias in the reported outcomes.

Study name	Randomization	Blinding	Withdrawal	Total score	Quality assessment
Al-Kaisy et al., 2018 [21]	2	2	1	5	High quality
Bolash et al., 2019 [22]	2	0	1	3	Low quality
De Andres et al., 2017 [23]	2	1	1	4	High quality
Hara et al., 2022 [24]	2	2	1	5	High quality
Kumar et al., 2007 [25]	2	0	1	3	Low quality
Rigoard et al., 2019 [26]	2	2	1	5	High quality

**Table 3:** Cochrane risk of bias assessment for the included studies The studies were evaluated based on key criteria including random sequence generation, allocation concealment, blinding of participants and personnel, blinding of outcome assessment, incomplete outcome data, selective reporting, and other sources of bias. Three studies demonstrated a low risk of bias and three studies demonstrated a moderate risk of bias.

Study name	Random sequence generation	Allocation concealment	Blinding of participants and personnel	Blinding of outcome assessment	Incomplete outcome data	Selective reporting	Other sources of bias	Overall risk of bias
Al-Kaisy et al., 2018 [21]	Low	Low	Low	Low	Low	Low	Low	Low
Bolash et al., 2019 [22]	Low	Low	High	High	Low	Low	Low	Moderate
De Andres et al., 2017 [23]	Low	Low	High	High	Low	Low	Low	Moderate
Hara et al., 2022 [24]	Low	Low	Low	Low	Low	Low	Low	Low
Kumar et al., 2007 [25]	Low	Low	High	High	Low	Low	Low	Moderate
Rigoard et al., 2019 [26]	Low	Low	Low	Low	Low	Low	Low	Low

Study characteristics: Characteristics of the sex and age of subjects included are summarized in Table 4. The included studies involved a total of 519 patients across six RCTs. Male patients comprised between 39.40% and 66.67%, while the proportion of female patients ranged from 33.33% to 60.60%. The mean age of participants across studies ranged from 47.90 years to 58.50 years with most studies having a relatively balanced gender distribution.

**Table 4:** Demographic characteristics of the study participants: sex and age distribution

Study name	Total number of patients	Total number of male patients	Total number of female patients	Percent of male patients	Percent of female patients	Mean age in years
Al-Kaisy et al., 2018 [21]	24	16	8	66.67%	33.33%	47.9
Bolash et al., 2019 [22]	72	38	34	53%	47%	58.5
De Andres et al., 2017 [23]	55	26	29	47.27%	52.73%	52.76
Hara et al., 2022 [24]	50	23	27	46%	54%	50
Kumar et al., 2007 [25]	100	43	57	43%	57%	49
Rigoard et al., 2019 [26]	218	86	132	39.4%	60.6%	53.9

Efficacy of SCS on Back Pain Secondary to FBSS

**Figure 3:**
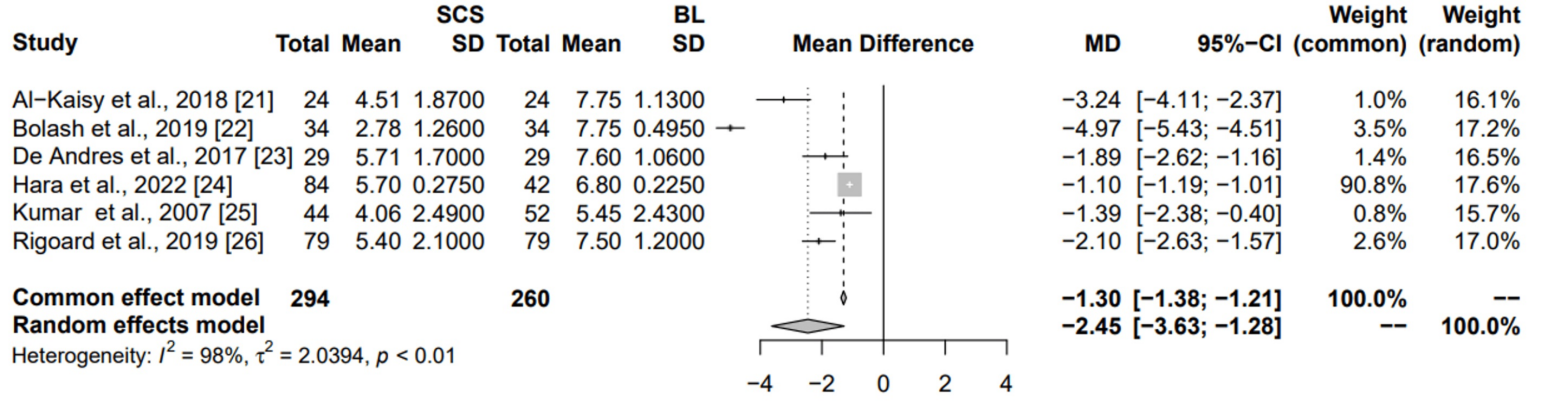
Summary of common and random effects for SCS vs baselines on chronic back pain secondary to failed back surgery MD: mean difference; CI: confidence interval; SD: standard deviation; SCS: spinal cord stimulation; BL: baseline

Comparison to the baseline: Six studies were examined to compare the effectiveness of SCS on back pain relief (Figure 3). Al-Kaisy et al. showed a significant reduction in pain scores with a mean difference of -3.24 (95% CI: (-4.11; -2.37)), contributing 1% to the common effects model and 16.1% to the random effects model. Bolash et al. demonstrated a substantial reduction in pain scores with a mean difference of -4.97 (95% CI: (-5.43; -4.51)), though with smaller weights of 3.5% (common) and 17.2% (random). De Andres et al., Hara et al., Kumar et al., and Rigoard et al. also reported reductions in pain scores, with varying degrees of mean differences and weights. The combined results from the common effects and random effects models indicate an overall reduction in pain scores due to SCS, with mean differences of -1.30 (95% CI: (-1.38; -1.21)) and -2.45 (95% CI: (-3.63; -1.28)), respectively. The high I² value of 98% suggests significant variability among the studies. Overall, this meta-analysis supports the effectiveness of SCS in reducing pain scores compared to the baselines.

**Figure 4:**
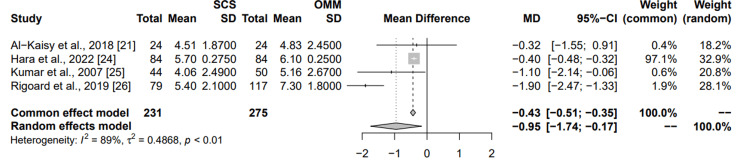
Summary of common and random effects for SCS vs OMM on chronic back pain secondary to failed back surgery MD: mean difference; CI: confidence interval; SD: standard deviation; SCS: spinal cord stimulation; OMM: optimal medical management

Comparison to the medical management: Four studies were chosen to compare the effectiveness of SCS versus optimal medical management (OMM) on back pain reduction (Figure 4). Hara et al. showed a reduction in pain scores with a mean difference of -0.40 (95% CI: (-0.48; -0.32)), contributing 97.1% to the common effects model and 32.9% to the random effects model. Al-Kaisy et al. and Kumar et al. also reported reductions in pain scores, with varying degrees of mean differences and weights. Rigoard et al. demonstrated a significant reduction in pain scores with a mean difference of -1.90 (95% CI: (-2.47; -1.33)), with weights of 1.9% (common) and 28.1% (random). The combined results from the common effects and random effects models indicate an overall reduction in pain scores due to SCS, with mean differences of -0.43 (95% CI: (-0.51; -0.35)) and -0.95 (95% CI: (-1.74; -0.17)), respectively. The high I² value of 89% suggests significant variability among the studies. This meta-analysis supports the effectiveness of SCS in reducing pain scores in back pain compared to OMM.

Efficacy of SCS on Leg Pain Secondary to FBSS

**Figure 5:**
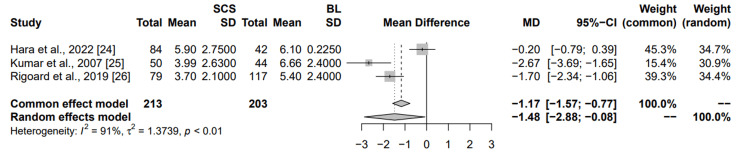
Summary of common and random effects for SCS vs baselines on chronic leg pain secondary to failed back surgery MD: mean difference; CI: confidence interval; SD: standard deviation; SCS: spinal cord stimulation; BL: baseline

Comparison to the baseline: Figure 5 illustrates the impact of SCS on leg pain scores compared to the baseline (prior to SCS implementation) across three studies. Hara et al. demonstrated that the mean difference in leg pain alleviation between SCS and OMM was -0.20 (95% CI: (-0.79; 0.39)), indicating a marginal, non-significant reduction in pain favoring SCS. Kumar et al. illustrated that the mean difference was -2.67 (95% CI: (-3.69; -1.65)), further confirming a significant reduction in leg pain favoring SCS. Rigoard et al. indicated that the mean difference was -1.70 (95% CI: (-2.34; -1.06)), revealing a statistically significant reduction in leg pain with SCS when compared to baseline. The pooled analysis utilizing both the common effects and random effects models indicated a statistically significant reduction in leg pain with SCS as opposed to baseline, with the common effects model yielding a mean difference of -1.17 (95% CI: (-1.57; -0.77)), which denotes a significant alleviation of leg pain with SCS, and the random effects model demonstrating a mean difference of -1.48 (95% CI: (-2.88; -0.08)), thereby reinforcing the efficacy of SCS in mitigating leg pain. The heterogeneity among the studies included was high, as evidenced by an I² value of 91% and a p-value <0.01, implying that the findings are consistent across the various studies and statistically significant. This meta-analysis substantiates the efficacy of SCS in diminishing pain scores associated with leg pain when contrasted with the baseline measurements prior to the initiation of SCS.

**Figure 6:**
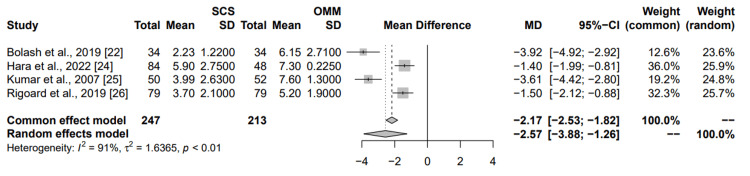
Summary of common and random effects for SCS vs optimal medical management on chronic leg pain secondary to failed back surgery MD: mean difference; CI: confidence interval; SD: standard deviation; SCS: spinal cord stimulation; OMM: optimal medical management

Comparison to the medical management: Four studies were incorporated into this meta-analysis assessing the comparative efficacy of SCS versus OMM in alleviating leg pain resulting from FBSS (Figure 6). Bolash et al. revealed a large reduction in pain scores of -3.92 (95% CI: (-4.92; -2.92)) with a smaller weight of 12.6% and 23.6% to the common effects and random effects model, respectively. Hara et al. showed a reduction in pain scores with a mean difference of -1.40 (95% CI: (-1.99; -0.81)), contributing a larger weight of 36% to the common effects model and 25.9% to the random effects model. Kumar et al. reported a mean difference of -3.61 (95% CI: (-4.42; -2.80)), with a 19.2% and 24.8% weight to the common and random effects model, respectively. Rigoard et al. also demonstrated a reduction in pain scores with a mean difference of -1.50 (95% CI: (-2.12; -0.88)), though with a moderate weight of 32.3% to the common effects model and 25.7% to the random effects model. The combined results from the common effects and random effects models both indicate an overall reduction in pain scores due to SCS, with a mean difference of -2.17 (95% CI: (-2.53; -1.82)) and -2.57 (95% CI: (-3.88; -1.26)), respectively. The heterogeneity statistics suggest significant variability among the studies, as indicated by the p-value <0.01. This meta-analysis elucidates that SCS is substantially more effective than OMM in diminishing leg pain in individuals afflicted with FBSS. The results are robust, characterized by high heterogeneity and consistent outcomes across the individual studies.

Discussion

The existing clinical trials concerning SCS as a therapeutic intervention for acute pain exemplify its efficacy; however, it is crucial to acknowledge that the scope of this research is confined to a singular study. This investigation indicates that SCS can markedly diminish pain scores and opioid usage within a brief timeframe. Notably, a singular session of SCS exhibited enduring effectiveness throughout the entire follow-up duration, which spanned six months. This observation parallels the one-time application utilized during surgical interventions, which retained efficacy for several weeks postoperatively until the conclusion of the follow-up period documented in a rodent model study [4]. In contrast to needing the implantation of SCS devices for the management of chronic pain, this finding represents a significant characteristic of SCS in the context of acute pain, indicating that the mechanism by which SCS operates in acute pain may inhibit its progression into a chronic condition. On the other hand, the effectiveness of SCS in alleviating chronic pain associated with FBSS has been comprehensively evaluated through multiple clinical trials, and its significance has been unequivocally validated [21-26]. The implementation of SCS for chronic pain management, particularly in individuals with FBSS, has demonstrated substantial analgesic benefits and enhancement of quality of life for these patients. The review encompassed multiple RCTs, illustrating that SCS can achieve a reduction in pain exceeding 50% in a notable proportion of patients with FBSS. Furthermore, SCS has been correlated with decreased opioid consumption and improved functional outcomes, rendering it a valuable modality for long-term pain management [27]. In conjunction with these examinations, the prospective utilization of SCS for the treatment of acute postoperative pain following spinal surgery signifies a burgeoning area of inquiry, particularly considering its well-established efficacy in alleviating chronic pain associated with FBSS.

Acute postoperative pain linked to spinal surgical interventions represents a substantial clinical challenge, often resulting in prolonged hospital stays, delayed recovery, and increased healthcare costs [6,28] along with the potential for progression into a chronic condition [29,30]. In addition, conventional pain management methodologies, such as opioids and regional anesthesia, are fraught with limitations, including adverse effects and inconsistent efficacy. SCS, which entails the application of electrical impulses to the spinal cord to modulate pain signaling, emerges as a potentially beneficial alternative. However, when juxtaposing the application of SCS for acute postoperative pain following spinal surgery with its use in chronic pain related to FBSS, several fundamental distinctions warrant careful consideration. The primary difference resides in the duration and nature of the pain experience among patients. Acute postoperative pain is characteristically transient and associated with surgical trauma, whereas chronic pain in FBSS is enduring and often neuropathic in nature [31,32]. The mechanisms underlying the action of SCS may also diverge between these two contexts. In the realm of acute postoperative pain, SCS likely functions by inhibiting nociceptive pathways and attenuating central sensitization [4]. In contrast, in the context of chronic pain, particularly in FBSS, SCS may exert its effects through more complex mechanisms involving both peripheral and central nervous system modulation [33].

The clinical implications of these findings are also of considerable importance. In the context of acute postoperative pain management, SCS could potentially reduce the reliance on opioids, thereby minimizing the risk of opioid-related side effects and complications. This is particularly relevant in the context of the ongoing opioid crisis. However, the implementation of SCS in the acute setting requires careful consideration of factors such as timing, patient selection, and cost-effectiveness. In the management of chronic pain associated with FBSS, SCS remains a cornerstone of management, and the robust evidence supporting its use underscores the importance of early referral and appropriate patient selection to maximize therapeutic outcomes. In addition, advances in SCS technology, including the development of high-frequency and burst stimulation, have further enhanced its efficacy and patient satisfaction. Nonetheless, complications and loss of efficacy pose a risk of SCS failure over time, with up to 10% of systems resulting in explanation at one year and up to 20% at five years [34-36]. While a washout period of months with residual pain control sometimes may be present, recurrence or worsening of chronic pain after explantation is expected in most patients [37].

In order to further advance the therapeutic approach of SCS, subsequent research should focus on conducting high-quality RCTs to evaluate the efficacy of SCS in acute postoperative pain across different surgical populations. The elucidation of the mechanisms by which SCS alleviates acute postoperative pain is also of paramount importance for academic investigation. Additionally, studies exploring the long-term outcomes and cost-effectiveness of SCS in this setting are warranted. Concerning chronic pain in FBSS, future studies should aim to refine patient selection criteria and optimize stimulation parameters to further improve outcomes.

## Conclusions

While its well-documented function in the management of chronic pain, especially in FBSS, establishes a robust basis for its extensive utilization, SCS demonstrates promising advantages for the alleviation of acute pain, as evidenced by both animal and human research studies. However, the evidence is still limited, and further high-quality RCTs are needed to establish the definitive role of SCS in this context. Continued research aimed at elucidating the intrinsic mechanisms of SCS in the realm of acute pain relief, alongside advancements in the optimization of SCS methodologies, will be crucial in unlocking the full potential of SCS in diverse pain management contexts.
